# Advances in Low Pt Loading Membrane Electrode Assembly for Proton Exchange Membrane Fuel Cells

**DOI:** 10.3390/molecules28020773

**Published:** 2023-01-12

**Authors:** Feng Cao, Rui Ding, Zhiyan Rui, Xuebin Wang, Zhen Meng, Bin Zhang, Weiwen Dong, Jia Li, Jianguo Liu, Xiangfen Jiang

**Affiliations:** 1 National Laboratory of Solid State Microstructures (NLSSM), Collaborative Innovation Center of Advanced Microstructures, College of Engineering and Applied Sciences, Nanjing University, Nanjing 210093, China; 2 School of Chemistry and Chemical Engineering, Nanjing University, Nanjing 210093, China; 3 Heraeus Precious Metal Technology (China) Co., Ltd., Nanjing 211511, China; 4 Institute of Energy Power Innovation, North China Electric Power University, Beijing 102206, China; 5 State Key Laboratory of Mechanics and Control of Mechanical Structures, Key Laboratory for Intelligent Nano Materials and Devices of the Ministry of Education, College of Material Science and Engineering, Nanjing University of Aeronautics and Astronautics, Nanjing 210016, China

**Keywords:** proton exchange membrane fuel cells, membrane electrode assembly, electrocatalysts, nanocatalysts, oxygen reduction reaction

## Abstract

Hydrogen has the potential to be one of the solutions that can address environmental pollution and greenhouse emissions from traditional fossil fuels. However, high costs hinder its large-scale commercialization, particularly for enabling devices such as proton exchange membrane fuel cells (PEMFCs). The precious metal Pt is indispensable in boosting the oxygen reduction reaction (ORR) in cathode electrocatalysts from the most crucial component, i.e., the membrane electrode assembly (MEA). MEAs account for a considerable amount of the entire cost of PEMFCs. To address these bottlenecks, researchers either increase Pt utilization efficiency or produce MEAs with enhanced performance but less Pt. Only a few reviews that explain the approaches are available. This review summarizes advances in designing nanocatalysts and optimizing the catalyst layer structure to achieve low-Pt loading MEAs. Different strategies and their corresponding effectiveness, e.g., performance in half-cells or MEA, are summarized and compared. Finally, future directions are discussed and proposed, aiming at affordable, highly active, and durable PEMFCs.

## 1. Introduction

**Scheme 1:**
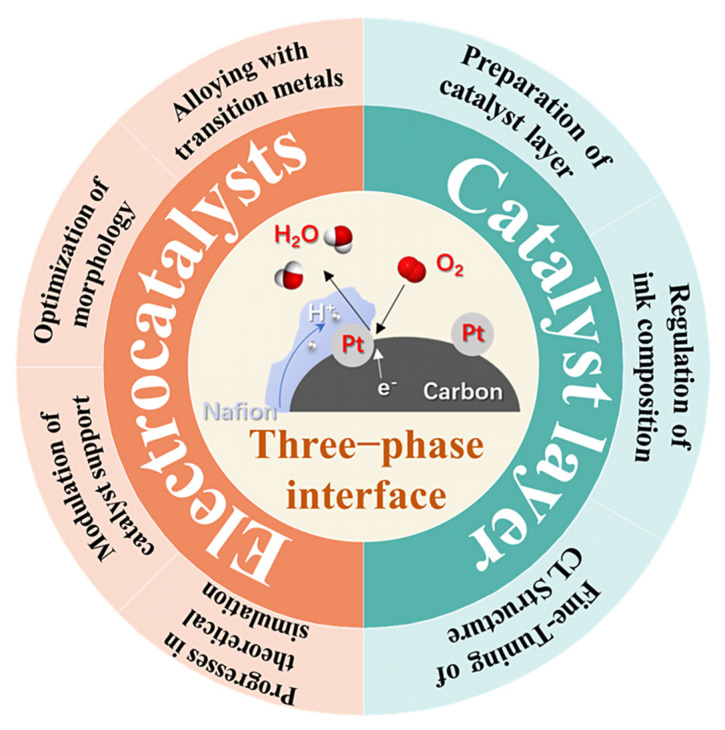
An overview of strategies for PEMFC cost reduction and performance improvement.

Energy demand is constantly increasing, and the accompanying environmental pollution and greenhouse effect are intensifying. Developing renewable energy technologies and promoting their commercialization are the keys to meeting energy demands while protecting the environment [1,2,3,4]. Hydrogen is one of the most promising forms of renewable energy because it has various forms of utilization and is an environmentally friendly product. Additionally, its advantages in safety, energy density, and storage simplicity are noteworthy [5]. However, its large-scale application is currently hindered by the high costs of devices that convert hydrogen [6,7,8]. Proton exchange membrane fuel cells (PEMFCs) are crucial for efficiently converting the chemical energy stored in hydrogen into clean electricity [9,10,11,12]. Hence, increasing the output power density and decreasing the cost of PEMFCs have become the goals of researchers in the field. These measures are shown in Scheme 1.

**Figure 1:**
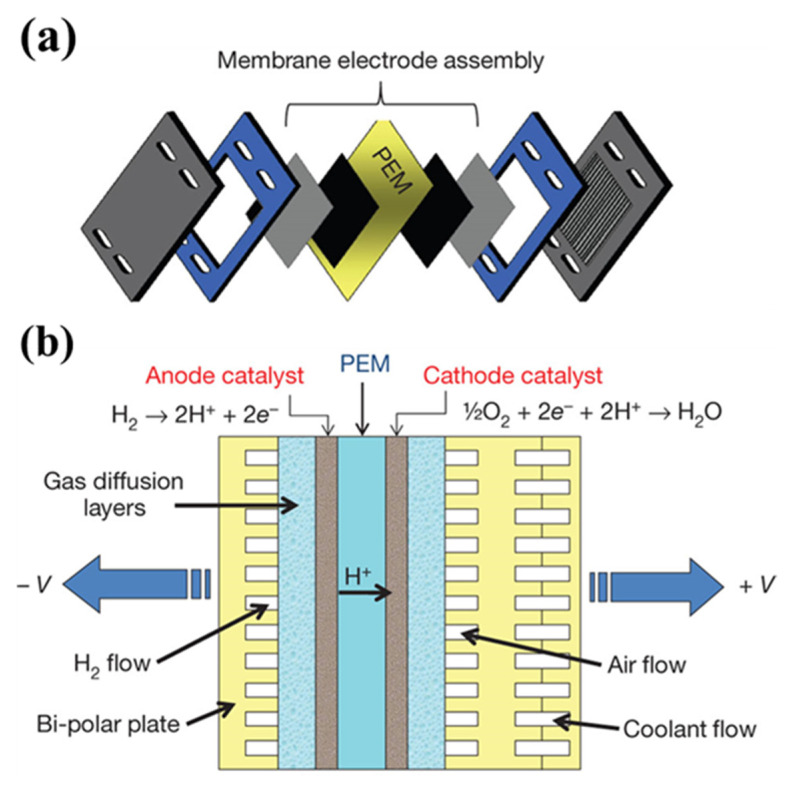
(**a**) Composition structure and (**b**) operational principle of PEMFC. Reprinted with permission from Ref. [13]. Copyright 2012, Springer Nature.

A single-cell PEMFC is shown in Figure 1a [13]. Its components include a flow field plate, a sealing layer, a gas diffusion layer (GDL), a catalyst layer (CL) on both sides of the anode and cathode, and a proton-exchange membrane (PEM) in the middle. GDL, CL, and PEM constitute the membrane electrode assembly (MEA), which is regarded as the most critical part of a PEMFC. The key electrochemical process of unleashing the chemical energy occurs in MEA, and the reaction speed determines the output power density in practical application. The so-called three-phase interface, where the gas, ionomer, and catalyst converge and where electrochemical reactions occur, is found in the CL. During PEMFC operation, electrochemical reactions occur simultaneously at both sides of the electrode interface. As shown in Figure 1b, H_2_ from the anode side first enters the flow field plate. It then passes through the GDL and goes inside the CL. A hydrogen oxidation reaction occurs at the anode electrocatalyst, producing protons and electrons. Protons are then transported through the PEM to the cathode side, and electrons travel through the external circuit. Similarly, O_2_ reaches the CL on the cathode side through a similar diffusion path and undergoes an oxygen reduction reaction (ORR) at the three-phase interface at the cathode electrocatalysts. The fuel cell continuously produces electricity with a continuous supply of hydrogen and air. Current research suggests that PEMFC performance is mainly determined by the activity of the cathodic catalyst and CL; a sluggish ORR is a rate-determining step.

Hence, many electrocatalysts (in most cases, Pt-based catalysts) are required to boost electrochemical reactions, increasing PEMFC manufacturing costs. Moreover, electrocatalyst stability and MEAs that can survive long-term service are critical for practical application. Therefore, optimizing the electrocatalyst and the CL structure (determining interfaces) to achieve minimum precious metal usage, high activity, and long-term stability is critical. Thus, PEMFCs require MEAs with low-Pt loading and with ideal output power density and durability. Researchers have proposed different strategies. Introducing additional elements [14,15,16], modulating the morphologies of electrocatalysts [17,18,19], and adjusting engineering parameters for CL [20,21,22,23] have been proven effective, and considerable progress has been achieved.

This review presents a summary of recent publications about low-Pt loading in MEAs for PEMFCs. Two perspectives on different scales are discussed: ORR electrocatalysts and the CL structure in MEA. The progress in ORR electrocatalysts is divided into three parts: nanocatalyst alloying, morphology design, and catalyst–support development. For CL structures, preparation methods, catalyst ink modulation, and unique CL structure designs are described. Furthermore, a comprehensive review is provided, ranging from enhancing microscopic electrochemical reactions to lowering mesoscopic mass transfer resistance and managing the multiphase flow of gas/water reactant/product. We also provide an outlook on future developments in durable electrocatalysts and CLs. This review is a valuable introduction for readers new to this field and a source of new insights and perspectives for experienced researchers.

## 2. Electrocatalysts for ORR in MEA

### 2.1. Alloying with Transition Metals

The widely adopted electrocatalyst for ORR is Pt nanoparticles loaded onto carbon supports. However, its catalytic activity is limited. Moreover, its stability is limited under harsh operating conditions due to the Ostwald ripening and particle migration processes [24]. Alloying can optimize the electronic structure of Pt nanoparticles and regulate their adsorption of oxygenated species, enhancing catalyst performance while reducing Pt loading [25,26]. Therefore, alloying Pt with other transition metals has been an important research direction in recent years [27]. Markovic et al. conducted a comprehensive study on the fundamental relationship between the surface electronic structure and ORR performance of two different Pt_3_M (M represents other transition metals) catalysts. They found that different compositions affect the electronic structure and alter the catalytic activity of the catalysts [28]. Pt_3_Co was found to exhibit the best specific activity based on the volcano-shaped relationship between the ORR catalytic activity and the corresponding descriptor of electronic structure, the d-band center, in Pt_3_M. Therefore, the study has proven that appropriate composition adjustment is a way to improve the ORR activity of PtM alloys.

**Figure 2:**
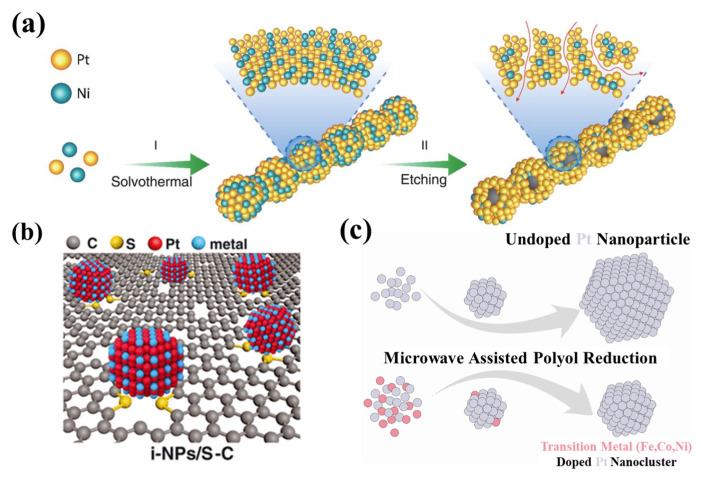
Catalyst performance enhancement through alloying, ordering, and doping: (**a**) Synthesis route and structure of PtNi alloy. Reprinted with permission from Ref. [30]. Copyright 2019, AAAS. (**b**) Diagram of the sulfur-anchored ordered alloy method. Reprinted with permission from Ref. [31]. Copyright 2021, AAAS. (**c**) Schematic of transition metal doping method. Reprinted with permission from Ref. [32]. Copyright 2022, John Wiley & Sons.

In previous studies, PtCo and PtNi alloy catalysts have shown great potential as substitutes for Pt in PEMFCs. Huang et al. used wet-chemical methods to prepare Pt_3_Co nanowires with abundant high-index surfaces enriched with Pt atoms and ordered structures [29]. Because of this unique structure, the catalyst is highly active in boosting ORR, with a mass activity (MA) and specific activity (SA) 33.7 and 39.6 times superior to commercial Pt/C, respectively. Furthermore, the catalyst composition and structure remain almost unchanged after 20,000 dynamic voltage cycles in the accelerated degradation test (ADT). The MA retains 91.9% of the initial value. Xia et al. prepared one-dimensional PtNi bunched nanospheres with different Pt and Ni ratios (Figure 2a) [30]. The final product, one-dimensional PtNi bunched nanocages, was synthesized using selective removal of nickel species under acidic conditions. The MA and SA of the best-performing catalyst are 3.52 A and 5.16 mA cm_Pt_^−2^, far exceeding those of commercial catalysts (0.21 and 0.36 mA cm_Pt_^−2^ for MA and SA, respectively). The catalyst activity did not decay considerably after 50 k dynamic voltage cycles in ADT, reflecting its excellent durability. The outstanding ORR performance of PtNi bunched nanocages should be attributed to the unique structure, the suitable Pt/Ni ratio, and the synergistic effects of stress and coordination. This finding provides new insights into the structural modulation of alloy catalysts.

Structurally ordered intermetallic compounds (IMCs), members of PtM catalysts, have been reported to be the most promising ORR electrocatalysts due to their unique properties [33]. By carefully controlling the structure of the catalysts to a highly ordered form, the unique IMC electronic structure remarkably improves the ORR catalytic performance [34]. By using structural and electrochemical characterizations, Mayrhofer et al. found that ordered, structured alloy catalysts exhibit superior activity and stability than disordered, structured ones [35]. However, their preparation usually involves a high-temperature annealing treatment. This treatment inevitably causes the IMC particles to grow in size, yielding low catalyst surface exposure. The result is limited performance on MEA in single-cell tests, despite promising activity observed in rotating disk electrodes (RDE) in half-cell tests [36]. To address this issue, Liang et al. employed a novel sulfur anchoring method (Figure 2b) [31]. This method takes advantage of the strong interaction between sulfur and platinum atoms to anchor the Pt-based nanoparticles on the carbon support like “solid glue.” The interaction prevents nanoparticle aggregation and enhances its catalytic durability. As a result, they were able to synthesize small-sized IMC catalysts and construct a material library consisting of 46 Pt-based binary and multiple IMC catalysts, which exhibit excellent MEA performance. In a later work, they further developed a synthetic strategy using heteroatom-containing molecules as additives in the impregnation process. A universal synthesis of 18 small-sized binary Pt IMC catalysts through the dual action of Pt-S chemical bonding and physical domain limiting could be achieved. The prepared PtCo catalysts exhibit outstanding MEA performance at low-Pt loading [37].

Doping is another way of improving the activity and durability of Pt alloy nanocatalysts. Li et al. synthesized ultrafine L1_0_-PtCo nanoparticles doped with a small number of metals [38]. The catalyst performance is remarkably better than that of commercial Pt/C catalysts. Moreover, excellent MEA activity and stability have been observed after 50 k dynamic voltage cycles at 80 °C. Extended X-ray absorption fine structure analysis and density functional theory (DFT) simulations have also indicated that W doping stabilizes the intermetallic structure and adjusts the Pt-Pt bond distance, thus weakening the adsorption of oxygen-containing species at the Pt surface. In another recently published work, Duan et al. prepared sub-2 nm Co-doped Pt nanoclusters using micro-wave assisted polyol reduction (Figure 2c) [32]. Unprecedented activity and stability were achieved from the as-prepared nanoclusters, mainly due to the extensive exposure of unique low-coordination sites on the surface of these nanoclusters. With a small amount of cobalt doping, the electrochemically active surface area (ECSA) of Pt nanoclusters is remarkably higher than that of undoped and commercial catalysts. Structural and compositional characterizations and DFT simulations show that the total energy of the nanoclusters decreases after doping. This decrease reflects nanocluster stabilization. Doping further reduces the d-band center and optimizes the binding energy of oxygen-containing intermediates, reducing the energy-determining step barrier and enhancing nanocluster activity. In real H_2_-air fuel cell evaluations, the catalysts easily outperform commercial Pt/C. More importantly, the commonly observed considerable performance loss in the high-current density region due to decreased Pt loading is substantially alleviated. In addition, doping also increases vacancies and improves nanocluster stability. As a result, the Co-doped nanoclusters have been proven to be more stable than a commercial catalyst in an ADT test of 30 k dynamic voltage cycles.

Currently, the effects of introducing additional transition metals are recognized as ligand and strain effects. The ligand effect refers to the direct electron transfer between Pt and the metal atoms, while the strain effect means lattice compression induced by nearby atoms with different atomic radii. These two effects shift the Pt d-band center downward and reduce the Pt binding energy and reaction intermediates, thus optimizing the ORR pathways [39]. However, the most serious problem faced by current Pt alloys is poor durability. Under high potential and strong acidic electrolytes during PEMFC operation, the introduced transition metals are susceptible to oxidation and dissolution. Additionally, the dissolved metal ions may cause ionomer and PEM degradation, resulting in poor MEA performance [40]. Fine-tuning the morphology of Pt alloy nanoparticles may be a possible solution. As a surface reaction, the geometry and structure of the catalyst influence the catalytic activity and stability. Therefore, besides modulating the Pt-based nanocrystal components, optimizing the morphology is also an effective strategy for increasing Pt-based catalyst efficiency [41].

### 2.2. Optimization of Morphology

**Figure 3:**
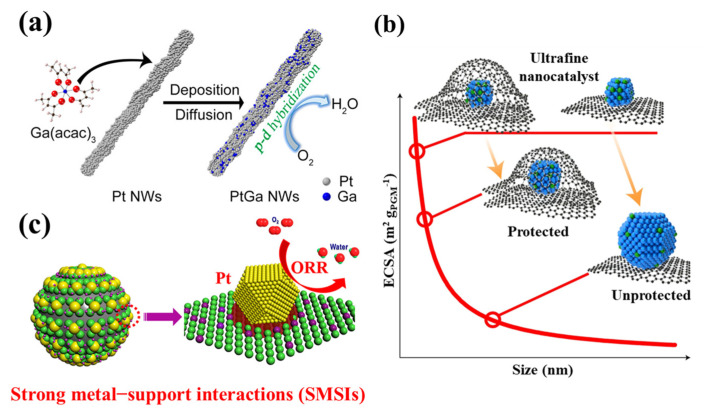
Catalyst performance enhancement through structural optimization: (**a**) Diagram of the design of PtGa NWs. Reprinted with permission from Ref. [48]. Copyright 2019, ACS. (**b**) Diagram showing the protective effect of special support on ultrafine catalysts. Reprinted with permission from Ref. [49]. Copyright 2022, Springer Nature. (**c**) Schematic of the SMSI effect between Pt and support. Reprinted with permission from Ref. [50]. Copyright 2018, Elsevier.

In recent years, researchers have designed and fabricated diverse Pt-based alloy geometry structures, including nanowires [42], nanoplates [43], nanoframes [44], and other special structures [45,46]. In general, increased active site exposure and accelerated electronic conduction afford excellent performance. Moreover, unique morphologies and enhanced crystallinity improve durability, making PEMFCs suitable for practical applications [47]. Huang et al. reported a novel catalyst based on PtGa ultrafine nanowires (Pt_4.31_Ga NWs/C), which exhibit strong p-d orbital hybridization interactions by doping with p-region metal elements (Figure 3a). This strategy inhibits component dissolution and precipitation of the alloy catalyst, thereby achieving high durability [48]. Notably, as a result, the Pt_4.31_Ga NWs/C catalyst shows only a 15.8% loss in MA after 30 k dynamic voltage cycles. In contrast, 79.6% are lost in commercial Pt/C under the same condition. DFT simulations show that the p-d orbital hybridization interaction between Pt and Ga atoms optimizes the surface electronic structure and improves the Pt oxidation resistance. Additionally, Ga dissolution is also inhibited, causing a remarkable improvement in ORR performance.

Particle size also affects electrocatalytic activity because ORR is a surface reaction. Small nanoparticles have high ECSA and expose many active sites under the same Pt loading, yielding a high MA [51]. However, catalyst particle size reduction brings a disadvantage in surface energy. They are more likely to agglomerate, causing a decrease in durability. However, this issue could also be mitigated using a delicate design of catalyst structures [52]. Huang et al. designed a specially structured catalyst in which a graphene shell encapsulates PtCo nanoparticles (Figure 3b). The nanocatalyst exhibits excellent electrochemical activity. Most impressively, it shows outstanding durability at an ultra-low-Pt loading of 0.07 mg_Pt_ cm^−2^ due to the noncontact shell of the graphene nanopocket [49]. With this design, catalyst particle aggregation and shedding are limited without affecting mass transport during ORR.

The surface properties of nanoparticles are closely related to their structure. Specifically, surface catalysis in transition metals depends mainly on the crystal facets. Furthermore, different nanocrystal morphologies afford varying degrees of exposure from different crystal facets, causing variation in efficiency and selectivity during catalytic processes [53]. Previous studies have reported that ORR kinetics at the Pt surface in 0.1 M HClO_4_ varies with the Miller index. The trend follows the order Pt (100) < Pt (111) < Pt (110) [54]. Therefore, modulating the catalyst structure to expose specific preferred facets is also a promising and important way to enhance ORR activity. For example, PtNi octahedral nanocrystals have a higher proportion of (111) facets than cubic nanocrystals; hence, their theoretical activity is higher than that of cubic nanocrystals. Xia et al. synthesized Pt_2.5_Ni octahedra to promote (111) facet formation and found that the activity of PtNi octahedra is remarkably enhanced due to the clean and well-preserved surfaces of the catalyst, with 17 and 51 times more MA and SA than commercial Pt/C, respectively [55]. Similarly, Huang et al. prepared highly dispersed PtNi octahedra on carbon supports, which improved surface exposure and catalytic activity. Good stability is observed after 6 k cycles of potential cycling [56]. Furthermore, the design of the core–shell structure can improve the utilization of Pt atoms and further decrease the amounts of precious metals. Because of this, ideal core–shell interactions can be obtained to enhance the performance of catalysts. Zeng et al. synthesized Pd@Pt_1.8_Ni octahedra with an ultra-thin alloy shell. The structure improves catalytic performance mainly due to the PtNi alloy and the interaction between the shell layer and the nucleus, which changes the electronic structure and atomic geometry [57]. Previous reports have proven that the high-index Pt crystal planes have better catalytic activity than other low-index crystal surfaces. Moreover, the core–shell structure with Pt skin reduces noble metal requirements while providing considerable catalytic performance. If the two structural features above can be combined into one morphology, the combined advantages might further promote electrocatalytic reactions [58,59]. Correspondingly, Guo et al. designed a novel synthetic route to obtain one-dimensional Pt_3_Fe nanowires with high-index facets and formed Pt-skin structures approximately two atomic layers thick on their surface [60]. The MA and SA of Pt_3_Fe nanowires are 17 and 13 times higher than those of commercial Pt/C, respectively. Moreover, excellent electrochemical stability is obtained. DFT simulations have revealed that the coordination and stress effects exhibited by this unique structure optimize the adsorption energy of oxygen-containing species, thus greatly increasing the catalytic performance.

The support material does not directly affect the overall performance; however, investigating it is still vital. Durability is a decisive factor in determining whether an electrocatalyst can be employed. Additionally, conductivity, which affects output power density, is closely related to the support type. Hence, designing support materials to meet the challenges of high potential is also an important topic to address.

### 2.3. Modulation of Catalyst Support

In addition to optimizing catalyst structure and composition, modulating support materials is also important for improving catalytic performance. Currently, the most commonly adopted catalyst–support combination in MEA is Pt-based nanoparticles loaded on a carbon support. The weak interaction between the metal particles and the carbon material is not strong enough for stabilization; therefore, metal particle migration and sintering are likely to occur, causing a remarkable decrease in long-term catalytic activity [33]. Moreover, carbon supports are vulnerable to oxidation and corrosion under harsh conditions, which can lead to metal particle detachment and agglomeration [61]. Introducing transition metal oxides as catalyst supports is a promising way. It can alleviate support corrosion and anchor the metal particles with strong metal–support interactions (SMSI) [62]. Liao et al. reported a novel high-performance catalyst (TiNiN@Pt), which shows only a slight loss of performance after 10 k dynamic potential cycles. Its structure also remained intact [63]. The excellent durability of the TiNiN@Pt catalyst is attributed to the good stability of the nitride and the synergy effect between Pt atoms and the stable TiN supports. In addition, Qiao et al. produced hybrid nanocatalysts by combining Pt nanoparticles with CoO nanorods on conductive carbon fiber paper substrates [64]. The electronic interaction between Pt nanoparticles and CoO nanorods and the direct growth of CoO nanorods on the carbon fiber paper substrate prevents the aggregation and separation of Pt nanoparticles, providing remarkably better performance than commercial Pt/C. Despite the excellent stability of metal oxides and the SMSI of metal particles, these materials have a drawback. Their limited electronic conductivity is insufficient to meet the targets for large-scale PEMFC applications.

Another way to enhance stability is by using highly graphitized nanocarbons as supports, such as carbon nanotubes, carbon nanocages, and graphene. Their oxidation and corrosion are less severe than those of traditional supports [65]. Li et al. prepared Pt/SH-CNT (carbon nanotube) catalysts with –SH groups on the surface of carbon nanotubes [66]. The strong interaction between Pt and S atoms causes a uniform distribution of Pt on the carbon nanotube surface while inhibiting Pt dissolution and agglomeration. Excellent electrochemical properties of the catalysts have been observed during performance evaluation. Despite the excellent electronic conductivity and stability of graphene as catalyst support, its pristine surface is not conducive to dispersing Pt nanoparticles uniformly, which implies the need for graphene surface functionalization to promote good Pt nanoparticles dispersion [65]. Ding et al. demonstrated the excellent stability of Pt nanoparticles on the surface of N-doped graphene by studying the structural and electronic properties of Pt nanoparticles on different kinds of graphene [67].

In order to address the disadvantages of carbon and noncarbon supports, researchers combined the two to form composite support. This approach may have great potential to improve catalyst durability [68]. Xiao et al. designed a TiO_2_-C composite support with dispersed TiO_2_ nanocrystals on the carbon surface and then prepared Pt/TiO_2_-C catalysts by photo-deposition method [69]. The TiO_2_ nanocrystals remarkably enhance the stability of carbon support under harsh conditions. Moreover, the SMSI between TiO_2_ nanocrystals and Pt nanoparticles accelerated the electron transfer process in the catalytic reaction. The Pt nanoparticles are firmly anchored, preventing migration and aggregation on the support, providing a remarkable improvement in the stability of Pt/TiO_2_-C catalysts. Similarly, Ye et al. used atomic layer deposition to deposit Ta_2_O_5_ nanoparticles onto carbon (Figure 3c). Pt nanoparticles were then deposited on this composite support. The support prevents Pt nanoparticles from detachment, migration, and agglomeration during PEMFC operations [50].

From the research works introduced above, it is fair to say that many highly active and durable catalysts for ORR have been developed. We summarized these catalyst properties (Table 1). Note that most of these results are based on half-cell tests on RDE. For practical applications, engineering and optimization of the CL structure are essential to obtaining high-performance MEA [70].

### 2.4. Progresses in Theoretical Simulation

Despite experimental explorations, theoretical simulations via DFT could help understand the catalytic mechanism and screen potential candidates. Norskov et al. first proposed to use free-energy changes in the adsorbed intermediate such as oxygen or hydroxyl, to describe the origin of ORR overpotential [71]. Additionally, the superiority of Pt in the volcano-shaped curve for ORR activity was explained by its moderate d-band center. Furthermore, they investigated the binary alloy system and explained the experimental observation of the excellent performance of Pt-Co/Ni/Fe from the DFT perspective [26]. Since then, researchers have extensively applied theoretical simulation as a powerful tool for explaining the origin of the ORR activity of electrocatalysts. It should be noted that, more importantly, in recent years, researchers have applied machine learning (ML) and artificial intelligence (AI) models to boost the high throughput screening for potential ideal material components and systems [72,73,74,75,76,77,78,79]. As surrogate models, as-trained ML models could significantly reduce the massive demand for computational resources by DFT calculations. For instance, Kang et al. trained a high-dimensional neural network potential based on a dataset of 13,877 DFT results for PtNi, PtCu, CuNi, and PtCuNi nanoparticles as a function of size and composition [80]. By combining Monte Carlo and molecular dynamics, the authors found a stable and active ternary nanoalloy electrocatalyst for ORR in acids: a 2.6 nm icosahedron containing 60% Pt and 40% Ni/Cu. Similarly, Rück et al. focused on Pt alloy nanoparticles with a core–shell structure [81]. They trained ML models as an alternative to the costly DFT calculations to predict the corresponding strains directly from geometrical structural features. The optimal size of Pt@Cu and Pt@Ni for ORR was modeled as 1.9 nm, while 2.8 nm was suitable for Pt@Au and Pt@Ag. More importantly, the authors demonstrated in the paper that the advantages of the ML model in terms of computational efficiency become superior as the size of the particles studied increases. Additionally, for the high entropy alloy system, which is considered a hot topic and more complex than binary/ternary alloys, such advantages have been proven by a series of works conducted by Rossmeisl and coworkers. They extensively built a workflow based on ML to reveal the ideal component in Ag-Ir-Pd-Pt-Ru and Ir-Pd-Pt-Rh-Ru systems finally. Such results could be impossible via traditional DFT simulation with astronomical computational costs. Therefore, ML might be a powerful novel tool in theoretically predicting potentially efficient catalysts that deserve attention.

## 3. CL Structure in MEA

### 3.1. Preparation of CL

**Figure 4:**
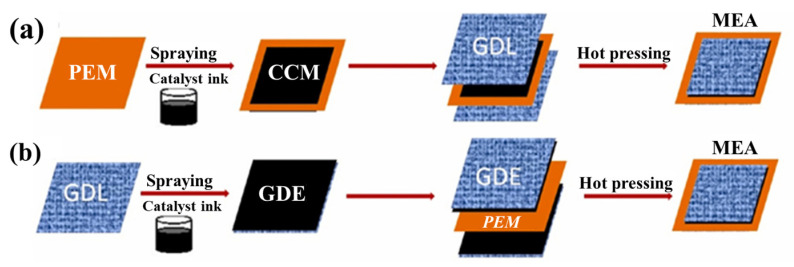
Preparation process of MEA: (**a**) CCM and (**b**) GDE methods. Reprinted with permission from Ref. [83]. Copyright 2021, Elsevier.

The core component of PEMFC is the MEA, where the cathode CL is where ORR occurs. The catalyst ink consists of a catalyst, an ionomer, and a solvent, which are then coated onto the GDL or PEM to form the CL. The ideal CL requires a high surface area catalyst/ionomer interface that boosts the reaction, a uniform ionomer distribution, and a suitable pore structure to facilitate proton, O_2_, and H_2_O transport. The structure of the PEM/CL interface has an important influence on the charge transfer and mass transfer processes in the MEA [82]. CL preparation can be divided into two types (Figure 4): gas diffusion electrode (GDE) and catalyst-coated membrane (CCM), which are distinguished by their different substrates. For the former, the catalyst ink is sprayed onto the GDL, and the surfaces are bonded together by a hot-pressing process. When the interfacial bonding disconnects, especially at the PEM/CL interface, local charge transfer stops, causing insufficient proton supply to assist the chemical reaction and thereby reducing catalyst utilization efficiency. Therefore, CCM preparation using PEM as the CL substrate is becoming more common to reduce adhesion failure at the PEM/CL interface [83]. Here, the catalyst ink is sprayed onto both sides of the PEM to improve bonding, making it less likely to detach and thus improving catalyst utilization [84].

**Figure 5:**
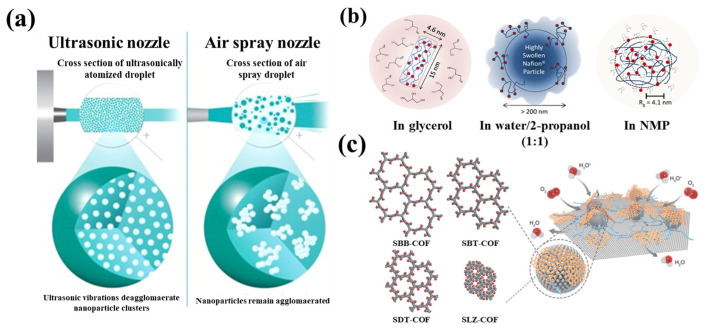
Modulation of spraying materials and methods to improve MEA performance: (**a**) Diagram of the ultrasonic spraying method. Reprinted with permission from Ref. [85]. Copyright 2010, Elsevier. (**b**) Dispersion of Nafion in different solvents. Reprinted with permission from Ref. [89]. Copyright 2012, ACS. (**c**) Modulation of the ionomer to optimize the structure of the three-phase interface. Reprinted with permission from Ref. [90]. Copyright 2022, AAAS.

Squeegee coating, screen printing, spray coating, and sputtering are generally used for CL formation. Ensuring thin-layer coating uniformity or large-scale production is challenging for these methods. In contrast, the ultrasonic spraying method (Figure 5a) is a novel next-generation approach for MEA preparation. Thin and uniform coatings are achieved using ultrasonic spraying, improving catalyst utilization and reducing MEA costs [85]. Pollet et al. believe that ultrasonic spraying technology can create special spraying conditions that form highly dispersed Pt nanoparticles, reducing agglomeration and providing high catalytic performance for ORR [86]. In addition, Eroglu [87] and Su [88] also find that MEAs prepared using the ultrasonic spraying technique exhibit excellent electrochemical properties.

### 3.2. Regulation of Ink Composition

The CL structure is closely related to the catalyst ink, especially the catalyst and ionomer dispersions in the solvent. These determine the three-phase interface, the distribution of ionomers, and the pore structure in the CL. Therefore, building the ideal CL structure by adjusting the ink composition and recipe is necessary [91]. The ratio of ionomer to carbon carrier (so-called IC ratio) is the first thing that can be associated with the modulation of catalyst inks. The ionomer content in the catalyst ink can have a remarkable impact on gas permeability and proton conductivity. When the ionomer content is too low, proton conduction is blocked, degrading electrochemical performance. However, when the ionomer content is too high, the ionomer blocks pore structures and hinders gas and water transport, also degrading electrochemical performance [92]. Lee et al. demonstrated, through a series of electrochemical performance evaluations, that the best activity and durability can be obtained at a moderate IC ratio of 1, whether the ink is water- or organic solvent-based [93]. In contrast, Yu et al. found that, for situations where Pt loading on MEA is relatively low, the appropriate IC ratio should be 0.3. This value changes with the support material [94]. In a recent study, Alink et al. employed two catalysts with different Pt loadings to test the gas and proton conductivity in the MEA under different conditions. They found that the optimal ionomer content correlates with both the Pt weight ratio in the catalyst and the Pt mass loading in the CL [95]. Therefore, the optimal IC ratio in the CL still needs to be investigated more deeply as an engineering issue in different cases. Advanced characterization methods are required to demonstrate its effect on the formed CL structure. Finally, water, proton, and gas conduction need to be evaluated.

The solvent also influences MEA performance. The solvent affects the size, viscosity, curing rate, and other properties of the ionomer particles in the catalyst ink. These then affect the newly formed CL structure [96,97]. Most studies choose water or ethanol as solvents; however, these media may not disperse ionomers well [98,99,100,101]. Hence, some researchers used mixed solvents for dispersion. Kim et al. noted that ionomers (e.g., Nafion) tend to form large particle network structures when dispersed in aqueous/alcoholic mixed solvents but form cylindrical rods or coil-like small particles when dispersed in polar solvents (Figure 5b) [89]. The chemical properties of the solvent molecules are the main reason for the varying structures. Uchida found that ionomers are dispersed uniformly in solvents with dielectric constants above 10 but form colloids between 3 and 10. Ionomers precipitate in solvents with dielectric constants below three [102]. However, the prevailing view is that the solvent influences the state of the ionomer; the relationship is still not thoroughly understood due to a lack of reliable approaches for characterizing the microstructure of catalyst inks [103].

Finally, ionomer properties also influence MEA performance. Park et al. analyzed CLs prepared from various ionomers containing different solvents [104]. They measured the thickness and contact angles of the ionomer films, tested the polarization curves, and investigated the high-frequency resistance of different samples. They found that the propylene glycol solvent in the ionomer yields small particles, allowing well-interconnected ionomers. Proton conduction is enhanced, resulting in excellent MEA performance. In another recent study, Zhang et al. incorporated ionic covalent organic framework nanosheets into Nafion to modify the interaction between the ionomer and the catalyst (Figure 5c). The approach yields a unique three-phase interfacial structure suitable for mass transfer in the CL. The MEA prepared using the composite ionomer exhibits good proton and gas transport and weakens –SO_3_H group adsorption on the Pt surface. These enhancements improve the Pt utilization, and the MA and peak power density in the MEA sample reach 1.6 times that of conventional MEA [90].

### 3.3. Fine-Tuning of CL Structure

**Figure 6:**
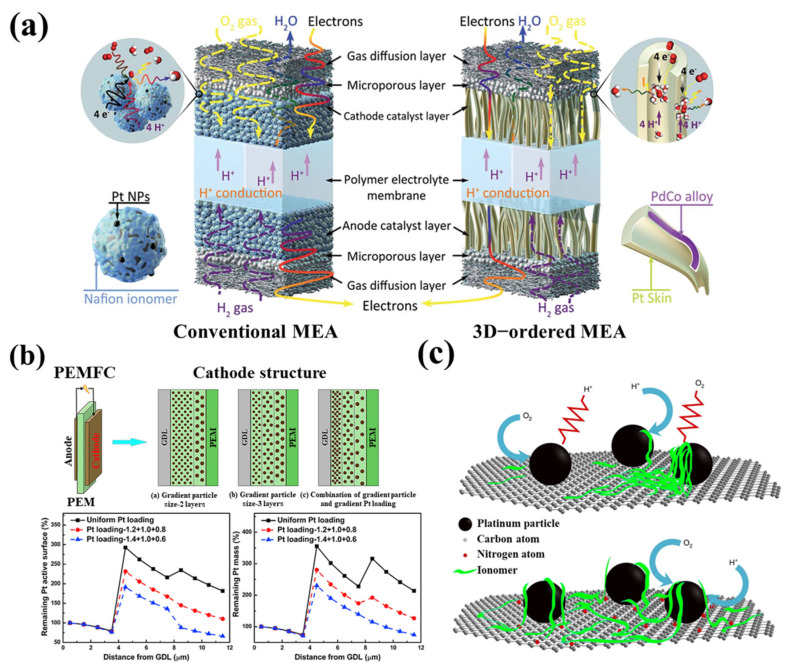
Optimization of CL structure to improve MEA performance: (**a**) Diagram of conventional MEA and ordered MEA. Reprinted with permission from Ref. [107]. Copyright 2018, RSC. (**b**) Design of Gradient CL Structure. Reprinted with permission from Ref. [114]. Copyright 2020, Elsevier. (**c**) Modified supports to regulate the distribution of ionomers. Reprinted with permission from Ref. [115]. Copyright 2020, Springer Nature.

In addition to modulating the catalyst ink composition, designing the CL structure can also be investigated. The CL can be fine-tuned to enhance electron, proton, water, and gas transport, thus achieving improved MEA performance. As mentioned previously, the two common ways of preparing CL are GDE and CCM. The latter has now become more commonly adopted because CLs with much lower interfacial impedance than those prepared using GDE can be obtained [105]. However, the ordered electrode (Figure 6a) developed in recent years is regarded as the future direction. In the ordered electrode, the transfer direction for electrons, protons, oxygen, and water is perpendicular to the PEM, enabling the maximum catalyst exposure. Mass transfer resistance is also reduced [106,107,108,109]. Vliet et al. developed nanostructured thin-film electrocatalysts based on this approach. The approach provides a large ECSA while reducing the contact resistance between the catalyst and the support, providing high catalyst utilization efficiency and low mass transfer resistance [110]. In addition, ordered CL structures based on carbon nanotube [111], carbon nanofiber [112], and polyaniline [113] arrays have also been investigated. These well-defined structures have proven effective in decreasing the dependence on precious metals while enhancing MEA performance.

CL design also involves adding some pore-forming materials and constructing a multi-stage CL. Zhao et al. reported uniformly dispersed catalysts with large surface pores and low gas diffusion resistance on the MEA surface by adding NH_4_HCO_3_ [116]. Chen et al. prepared CL-containing electrocatalysts with different Pt mass fractions and different ionomer contents to improve MEA catalyst utilization [117]. After electrochemical characterization, they found that intentionally placing more catalysts and ionomers near the PEM provides smaller ohmic and charge transfer resistances, causing excellent performance. Similarly, a bilayer CL structure with a gradient distribution of Pt content and pore structure was proposed by Ye et al. [118]. By changing the Pt and C contents, fabricating the CL near the PEM side, which has a high Pt mass loading, to promote the ORR reaction is possible. The CL near the GDL side should have a large pore size that can facilitate gas and water transport. This “allocation on demand” measure is able to decrease the amount of Pt effectively. Such an idea could be called the design of gradient structures. Investigations aimed at ionomer and catalyst particles are also conducted. Holdcroft et al. developed and evaluated GDEs with ionomer (Nafion) gradient distribution [119]. The results show that cathode performance is improved by placing more ionomers near the CL/PEM interface at a medium- to high-current density area. On the contrary, when the ionomer content gradient was reversed, the performance of the cell decreased remarkably. Unhindered proton transport near the CL/PEM interface must be ensured and high ionic conductivity in this region is required. Zhang et al. designed a highly durable MEA by combining particle size gradients in the CL structure with Pt loading (Figure 6b) [114]. By using mathematical models, they evaluated several gradient CL structures of Pt/C catalysts based on ECSA evolution and Pt mass during the cycling process. They found that Pt utilization and MEA performance could be improved by optimizing the particle size and the Pt loading gradient distribution. Therefore, a reasonable gradient design of the CL structure improves MEA catalytic activity. In addition, ionomer distribution in the CL can be improved by using functionalized carbon supports. Ott et al. obtained N-doped carbon supports by pre-oxidation and high-temperature ammonolysis processes (Figure 6c), which allowed uniform distribution of ionomers on their surfaces and reduced mass transfer resistance [115]. The pore structure of the porous carbon support was also optimized so that Pt nanoparticles were uniformly dispersed on the outer surface of the support. Excellent MEA performance was obtained. We summarized the performance of MEAs from related publications in Table 2 to reflect the influence of CL design on MEA performance.

## 4. Summary and Outlook

Recently, remarkable progress has been made in reducing PEMFC cost by improving the intrinsic activity of catalysts and optimizing the structure within the CL. In this review, we summarized and discussed the developments in catalysts and CL structures in recent years. For catalysts, alloying is the most common strategy to increase activity. However, catalyst stability is crucial; thus, studies focus on optimizing catalyst morphology. In addition to these, corrosion of the carbon support can occur under harsh conditions, leading to agglomeration and the shedding of catalyst nanoparticles. These changes then lead to activity loss and device degradation. Thus, further work on catalyst supports is essential to break through this bottleneck. Balancing catalytic activity, conductivity, and durability requires novel electrocatalyst designs. Furthermore, large-scale CL studies are also needed for PEMFC to be employed in practical applications. CL is not only the place where an electrochemical reaction takes place but also involves the transport of oxygen, electrons, protons, and water. Therefore, its influence on PEMFC performance is wide-ranging. Researchers proposed adopting modulation of the catalyst ink composition or designing the CL structure to obtain a reasonable three-phase interface that enhances Pt utilization and reduces Pt loading without sacrificing the performance of MEA.

Despite the current progress, there is still a long way to go in the commercialization process of PEMFCs. Novel catalysts and CLs designed for scientific research often encounter problems in practical applications. Thus, current catalysts and MEA structures used in most commercial power reactors are still conventional. For this reason, we propose several directions that might be able to bring a real change in the current situation.

1. In many cases, high activity or Pt utilization efficiency for the catalyst on RDE does not translate to good catalytic performance in MEAs [59,70,120]. This difference is due to different working conditions. Numerous studies continue to focus on RDE electrocatalyst performance due to their low cost. We suggest that electrocatalysts be evaluated and optimized in MEA and single cells. The findings would then be more convincing and valuable for commercial development. Low-Pt loading in MEA needs to be conducted for both catalyst and CL structures. The most pressing issue is MEA durability.
2. In developing low-Pt loading MEAs, a large ECSA is needed to address mass transfer issues in current catalysts. Therefore, catalyst particle size needs to be reduced [32,49,52]. However, this reduction causes migration and ripening, leading to reduced durability, and durability is sometimes more important than activity [121,122,123]. Currently, many studies still focus on obtaining enhanced catalytic activity. Stability is rarely the goal. ADTs are often performed after the best sample is chosen at the end. We suggest that researchers consider stability as equally important as activity in their research and development.
3. Nonprecious metal electrocatalysts have recently been proven as an alternative to traditional Pt-based electrocatalysts, even in MEA [124,125,126,127]. However, the stability of nonprecious metal electrocatalysts could not meet the demands of practical applications. Additionally, activity and stability are still significant challenges, especially in larger-scale application circumstances, such as in actual stacks [128,129]. Like the Pt-based material system that has been discussed, most studies of nonprecious metal-based electrocatalysts are still on RDE [72]. However, recent reports have indicated that combining nonprecious and precious metal catalysts may benefit from a synergistic effect [130,131,132]. This integrated field might be promising for enhancing ORR catalytic performance while keeping costs down.
4. The problem of mass transport severely affects the performance of low-Pt loading MEA [133,134,135,136]. Therefore, for CL, a good structure suitable for mass transfer needs to be developed. However, a cheap and effective solution has not yet emerged. Hence, in-depth studies focusing on suitable pore structures and homogeneous three-phase interfaces are needed.
5. It should also be noted that the reduction in noble metal loadings is not only important. Considering the limited crustal reserves, it is very economical to recycle precious metals from obsolete and defunct MEAs [137,138]. Specific manufacturing routes of the MEAs may have used less Pt but would increase the difficulties in recycling. Most researchers have not yet realized that the cost should eventually be “life-long”. Hence, it is suggested that such prospects be concerned in the future.

## Figures and Tables

**Table 1:** ORR performance of catalysts in the relevant literature.

Catalyst	Specific Activity (mA cm^−2^)	Mass Activity (A mg_Pt_^−1^)	Electrochemically Active Surface Area (m^2^ g_Pt_^−1^)	Accelerated Durability Test	Mass Activity Retention	Electrolyte	Reference
Au– Pt–Co/C-0.015	0.535	0.386	72.2	0.6–1.0 V 30 k cycles	90.6%	0.1 M HClO_4_	[14]
Pt_3_Co/NC	1.236	0.382	31.0	0.6–1.0 V 30 k cycles	104.5%	0.1 M HClO_4_	[15]
Pt–Ni–Au NWs	2.59	0.651	25.1	0.6–1.0 V 30 k cycles	82.3%	0.1 M HClO_4_	[16]
700- Pt1Co1-IMC@Pt/C-2.5	1.11	0.53	43.5	0.6–1.1 V 30 k cycles	76.6%	0.1 M HClO_4_	[17]
Pt/TiN@C		0.155	69.2	1.2 V constant for 400 h	95.9%	0.1 M HClO_4_	[18]
Ni_4_Co_2_Pt/CNFs Ni_6_Pt/CNFs	NA	35.7 A/g @ 799 mV for ethanol 37.4 A/g @ 799 mV for ethanol	NA	0.8 V constant chronoamperometry	57.4% (1000 s for methanol) 70.8% (1000 s for urea)	1 M KOH	[27]
Pt_3_Co NWs/C	7.12	3.71	52.1	0.6–1.1 V 20 k cycles	91.9%	0.1 M HClO_4_	[29]
PtNi BNCs/C	5.16	3.52	68.2	0.6–1.1 V 50 k cycles	98.7%	0.1 M HClO_4_	[30]
PtCu_3_	3.8	4.18				0.1 M HClO_4_	[31]
Co-doped Pt	0.498	0.579	116.2	0.6–1.0 V 30 k cycles	91.0%	0.1 M HClO_4_	[32]
PtCo	3.21	2.25	70.1			0.1 M HClO_4_	[37]
L1_0_-W-PtCo/C	3.60	2.21	61.4	0.6–1.0 V 10 k cycles	86.4%	0.1 M HClO_4_	[38]
PtGa NWs/ C	3.28	1.89	53.4	0.6–1.1 V 30 k cycles	84.2%	0.1 M HClO_4_	[48]
PtCo@Gnp	1.62	1.19	68.7			0.1 M HClO_4_	[49]
Pt/N-ALDTa_2_O_5_/C		0.28	70.3	0.6–1.0 V 10 k cycles	90%	0.1 M HClO_4_	[50]
Pt_2.5_Ni/C	7.3	3.3	45	0.65 V (5 s) 0.95 V (5 s) 5 k cycles	60%	0.1 M HClO_4_	[55]
PtNiCo/C	3.88	2.33	61.6	0.6–1.1 V 6 k cycles	43.3%	0.1 M HClO_4_	[56]
Pd@Pt_1.8_Ni		0.79	178.01	0.6–1.05 V 6 k cycles	NA (MA retention) 90% (ECSA retention)	0.1 M HClO_4_	[57]
Pt-skin Pt_3_Fe z-NWs/C	4.34	2.11	34.0	0.6–1.1 V 50 k cycles	75.4%	0.1 M HClO_4_	[60]
PtCo/TiO_2_/CNT	0.628	0.476	75.8	0.6–1.0 V 30 k cycles	88.8%	0.1 M HClO_4_	[62]
TiNiN@Pt	0.49	0.83	55.4	0.6–1.05 V 10 k cycles	91.6%	0.1 M HClO_4_	[63]
Pt/TiO_2_-C		0.21	81.7	0.6–1.0 V 10 k cycles	99.1%	0.1 M HClO_4_	[69]

**Table 2:** Properties of MEAs with different structures and components.

Catalyst	Cathode Pt Loading (mg_Pt_ cm^−2^)	Test Conditions	Peak Power Density (W cm^−2^)	Area (cm^2^)	Mass Activity (A mg_Pt_^−1^)	Accelerated Durability Test	Mass Activity Retention	Reference
Pt–Ni–Au NWs	0.1	H_2_/O_2_ 200/200 sccm 100 kPa_abs_	0.714 A cm^−2^ @ 0.6 V	1		0.6 V (3 s) 0.95 V (3 s) 10 k cycles	0.520 A cm^−2^ @ 0.6 V	[16]
Pt1Co1- IMC@Pt/C	0.2	H_2_/O_2_ 1000/400 sccm 100 kPa_abs_	1.45 A cm^−2^ @ 0.65 V	6.25	0.18	0.6 V (3 s) 0.95 V (3 s) 30 k cycles	75.2%	[17]
PtNi BNCs/C	0.15	H_2_/Air 150/300 sccm 30 psi	1.5 A cm^−2^ @ 0.6 V	5				[30]
PtCo	0.02	H_2_/O_2_ 200/200 sccm 150 kPa_abs_		5	1.52	0.6–1.0 V 30 k cycles	77%	[31]
PtCo	0.125	H_2_/Air 250 kPa_abs_	1.17		1.08	0.6 V (3 s) 0.95 V (3 s) 30 k cycles	75%	[37]
L1_0_-W-PtCo/C	0.11	H_2_/O_2_ 200/500 sccm 1.5 kPa_abs_			0.57	0.6–1.0 V 30 k cycles	82.5%	[38]
PtCo@Gnp	0.07	H_2_/O_2_ 835/2000 sccm 150 kPa_abs_	1.01	5	1.21	0.6 V (3 s) 0.95 V (3 s) 30 k cycles	73%	[49]
Sigracet SGL 10BC GDL	0.15	H_2_/O_2_ (stoichiometry 1.3/2.2) 200 kPa_abs_	0.53	16				[86]
Pt/C@SDT-Nafion	0.07	H_2_/Air 150 kPa_abs_	1.08	5	0.35	0.6 V (3 s) 0.95 V (3 s) 30 k cycles	62%	[90]
RA1 MEA		H_2_/Air (stoichiometry 1.5/3) 100 kPa_abs_	1.3 A cm^−2^ @ 0.6 V			0.6–1.0 V 30 k cycles	54.3% (ECSA retention)	[93]
Pt/Vulcan (Cabot)	0.07	H_2_/Air 500/1000 sccm	0.91	25	0.23			[94]
Pt_skin_@PdCo NTAs-400	3.5 μg_Pt_ cm^−2^	H_2_/O_2_ 100/200 sccm 200 kPa_abs_	0.78	2.56		0.6–1.0 V 5 k cycles	63.5% (power density retention)	[107]
Pt/N-KB	0.11	H_2_/Air 1000/2000 sccm 230 kPa_abs_	1.39	1.4	0.202	0.6–1.0 V 1.5 k cycles	98.5%	[115]
Gradient design MEA	0.2	H_2_/Air stoichiometry 1.5/2.5 100 kPa_abs_	0.69	25				[117]
Gradient design MEA	0.09	H_2_/Air 100 kPa_abs_	0.48	6.25				[118]

## Data Availability

Not applicable.
